# The impact of assisted reproductive technology treatment coverage on marriage, pregnancy, and childbirth in women of childbearing age: an interrupted time-series analysis

**DOI:** 10.1186/s12889-023-16286-3

**Published:** 2023-07-18

**Authors:** Il Yun, Wontae Cha, Chung-Mo Nam, Jin Young Nam, Eun-Cheol Park

**Affiliations:** 1 Department of Public Health, Graduate School, Yonsei University, Seoul, Republic of Korea; 2 Institute of Health Services Research, Yonsei University, Seoul, Republic of Korea; 3 CHA Hollywood Presbyterian Medical Center, Los Angeles, CA United States of America; 4 Department of Preventive Medicine, Yonsei University College of Medicine, Yonsei-ro 50-1, Seodaemun-gu, Seoul, Republic of Korea; 5 Department of Healthcare Management, Eulji University, Sanseongdae-ro 553, Sujeong-gu, Seongnam, Gyeonggi-do Republic of Korea

**Keywords:** Birth rate, Assisted reproductive technology, Infertility, Insurance, Policy

## Abstract

**Background:**

Korea is encountering major challenges related to its declining birth rate and aging population. Various policies have been introduced to prevent further population decrease and boost the birth rate, but their effectiveness has not been verified. Therefore, this study examined the effects of assisted reproductive technology (ART) insurance coverage on marriage, pregnancy, and childbirth in women of childbearing age.

**Methods:**

All information on marriage, pregnancy, childbirth of women of childbearing age was obtained from Statistics Korea and Korean National Health Insurance Service database. During a total follow-up period of 54 months (July 2015 to December 2019), an average of 12,524,214 women of childbearing age per month, and 29,701 live births per month were included in the analysis. An interrupted time series with segmented regression was performed to analyze the time trend and changes in outcomes.

**Results:**

The implementation of ART coverage policies had no significant impact on marriage or pregnancy rates. However, it did affect multiple pregnancy and multiple birth rates, which increased by 1.0% (Exp(β3) = 1.010, P-value = 0.0001) and 1.4% (Exp(β3) = 1.014, P-value = < 0.0001), respectively, compared to the pre-intervention period. Although the effect of covering ART treatment on total birth rates were not confirmed, a slightly slower decline was observed after the intervention (Exp(β1) = 0.993, P-value = < 0.0001, Exp(β1 + β3) = 0.996 P-value = 0.012).

**Conclusion:**

This study identified the effects of ART health insurance coverage policy on the rates of multiple pregnancies and births. After the policy implementation, the downward trend in the total birth rate reduced slightly. Our findings suggest that interventions to support infertile couples should be expanded to solve the problem of low fertility rates.

**Plain english summary:**

To address the intricate problems related to low birth rates, the Korean government introduced a policy that provides financial support and health insurance coverage for assisted reproductive technology (ART) treatment for infertile couples. As a result of evaluating the effectiveness of the ART coverage policy, it led to higher rates of pregnancies and births. In addition, although the total birth rate has been continuously decreasing over time, the decline may have been slowed down slightly by this policy.

**Supplementary Information:**

The online version contains supplementary material available at 10.1186/s12889-023-16286-3.

## Background

The average total fertility rate (TFR), which represents the average number of births per fertile woman, has decreased significantly in recent decades [1, 2]. As of 2021, the TFR of the Organization for Economic Cooperation and Development (OECD) countries was 1.63 [3], while Korea’s TFR was only 0.808, thereby ranking at the bottom for decades [4]. According to the United Nations (UN), countries with a TFR of 2.1 or less are considered low fertility, and those with a TFR of 1.3 or less are considered ultra-low fertility [5]. Korea was classified as a low-fertility country in 1983 and an ultra-low-fertility country in 2002 [4]. It is currently one of the fastest-aging nations in the world [6]. As of 2020, the number of deaths and births in Korea has reversed, leading to a decline of the population [4].

Korea is encountering major challenges related to its declining birth rate and increasing aging population. Diverse strategies are being tested to prevent further population decrease and boost the TFR, but their effectiveness has not been verified. To address the intricate problems related to low fertility rates, the Korean government introduced a policy that provides financial support and health insurance coverage for assisted reproductive technology (ART) treatment for infertile couples [7, 8]. Consequently, over the past two decades, the number of twin births in Korea has risen significantly from 5 pairs per 1,000 births in 2000 to 18 pairs per 1,000 births in 2019 [9]. Nonetheless, it remains unproven whether national policies that support ART actually contribute to an increase in national pregnancy and total birth rates.

Numerous studies have explored the effects of ART policies on fertility and reproductive outcomes. A US study analyzing the impact of introducing an ART insurance mandate found that the mandate led to a significant increase in the number of ART cycles performed and, consequently, in birth rates among women that underwent ART [10]. Additionally, some studies have reported that ART insurance coverage has increased the utilization of ART by women with infertility. However, this also results in an increase in the number of multiple births, which is a potential complication of ART [11, 12]. On these grounds, many developed countries support the cost of ART treatment according to the medical necessity for infertility, and some countries have come to provide support for treatment costs to infertile couples with the goal of national population policy to improve the fertility rates. For example, Japan government provide public subsidies for diagnosis and treatment of infertility, in vitro fertilization, and other assisted reproductive procedures in order to address the serious problem of low fertility and aging population [13].

Similarly, Korea also introduced a health insurance coverage policy for ART treatment in October 2017, but it has not yet been evaluated whether this intervention is effective for marriage, pregnancy, and childbirth in the country. Therefore, this study aimed to identify trends and changes in marriage, pregnancy, multiple pregnancies, multiple births, and total birth rates before and after the implementation of the ART health insurance coverage policy in Korea.

## Methods

### Data and study population

Population data from Statistics Korea and delivery cohort data from the Korean National Health Insurance Service (NHIS) were combined and used for analysis. Statistics Korea publishes population trends every month; therefore, the number of women of childbearing age, newly married women, pregnant women, and the total number of live births were identified using these data. The NHIS provides customized cohort data for academic research and policymaking. The NHIS delivery cohort data we obtained included information on all women who gave birth at Korean medical institutions between July 1, 2015, and December 31, 2019. The monthly number of multiple pregnancies and births was determined using the International Classification of Diseases, 10th Revision (ICD-10) diagnostic codes included in the claims data.

During the total follow-up period of 54 months, an average of 12,524,214 women of childbearing age and 29,701 live births per month were included in the analysis. The study protocol was reviewed and approved by the Institutional Review Board of Eulji University (IRB Number: EU22-27). The requirement for informed consent was waived because the Statistics Korea and NHIS databases do not contain any personally identifiable information.

### Variables

The variable of interest was whether ART treatment was covered by health insurance, which was divided into pre- and post-intervention periods. As the Korean NHIS introduced the ART coverage policy on October 1, 2017, and the last follow-up date in the data was December 31, 2019, we set the period before and after the intervention to 27 months. Therefore, the pre-intervention period was from July 1, 2015, to September 30, 2017, and the post-intervention period was from October 1, 2017, to December 31, 2019.

The dependent variables were as follows: First, marriage rates were calculated on a monthly basis as the percentage of newly married women of childbearing age. Second, pregnancy rates were calculated as the percentage of women of childbearing age who successfully conceived per month. Similarly, for the third and fourth outcome variables–multiple pregnancy rates and multiple birth rates–the denominator was set to the total number of women of childbearing age per month, and the numerator was set to the number of women with multiple pregnancies and the number of mothers with multiple births, respectively. Multiple pregnancies were identified using the ICD-10 code O30 and defined as cases in which two or more fetuses were conceived. Multiple births were determined using the ICD-10 code O84 and defined as cases in which two or more babies were born. Finally, the total birth rate was calculated as the ratio of the number of new births to the number of women of childbearing age per month.

### Statistical analysis

To analyze the time trend and its change in outcomes, we conducted an interrupted time series (ITS) with segmented regression. The ITS was modeled using a linear regression model that included three time-related variables, and the regression coefficients estimated the slope before intervention, the level change at the time of intervention, and the slope change after the intervention. A measurement of the difference between the slope before and after the intervention can be determined by the slope change. Level change indicates an absolute change in the level of outcomes at the time of intervention; that is, the immediate effect of the intervention [14]. As the log-link function was applied to the generalized linear model to perform the segmented regression, the model coefficients were converted into exponentials to represent the trends and changes in the outcomes on the original scale. In other words, to provide interpretations for the original scale, where E(Y_i_) = *µ*_i_, it is necessary to convert Log[E(Y_i_)] into multiplicative coefficients.

$$\begin{array}{c}\log \,({\mu _i}) = {\beta _0} + {\beta _1}\,*tim{e_t} + {\beta _2}\,*interventio{n_t}\\+ {\beta _3}\,*time\,after\,interventio{n_t} + {e_t}\end{array}$$

In this model, the intercept *β*_*0*_ estimates the baseline level of the outcome, *β*_*1*_ estimates the baseline trend of the outcome, *β*_*2*_ estimates the immediate effect size of the intervention by indicating the level change after intervention, and *β*_*3*_ estimates the change in trend after intervention. The sum of *β*_*1*_ and *β*_*3*_ is the slope after intervention, which indicates the follow-up outcome trend [15]. The results are presented as parameter estimates, standard errors, and *P*-values, with statistical significance defined as *P*-values less than 0.05. All analyses were performed using the SAS software (version 9.4; SAS Institute Inc., Cary, NC, USA).

## Results

Table 1 shows the monthly average number of variables used to calculate the outcome variables. The structure of the aggregated data used for the analysis is presented in Supplementary Table 1. During 54-month study period, the monthly average number of childbearing women was 12,524,214, and it can be seen that the fertile population decreased slightly after intervention compared to before intervention. Similarly, the number of married women who became pregnant and gave birth has declined over time.

**Table 1:** The monthly average number of variables used to calculate the outcome variables

	Average number of women of childbearing age per month	Average number of newly married women per month	Average number of pregnant women per month	Average number of mothers with multiple pregnancy per month	Average number of mothers with multiple birth per month	Average number of births per month
ART health insurance coverage						
Before intervention (2015.07 ~ 2017.09)	12,739,913	23,093	38,937	447	320	33,122
After intervention (2017.10 ~ 2019.12)	12,308,515	20,976	30,847	425	278	26,280
**Total**	12,524,214	22,034	34,892	436	299	29,701

Table 2 presents the results of the segmented regression analysis used to predict five outcome variables. Implementing the ART coverage policy had no statistically significant effect on marriage or pregnancy rates. However, the effects of the intervention were found in multiple pregnancies and multiple birth rates. Compared to before the intervention, the multiple pregnancy rates increased by 1.0% (Exp(*β*_*3*_) = 1.010, *P*-value = 0.0001) and the multiple birth rates by 1.4% (Exp(*β*_*3*_) = 1.014, *P*-value = < 0.0001) after the intervention, and both outcomes, which had decreasing trends, changed to increasing trends. Finally, the effect of ART coverage policies on the total birth rate was not confirmed. Although still on downward trend, the slope has flattened slightly as the intervention (Exp(*β*_*1*_) = 0.993, *P*-value = < 0.0001, Exp(*β*_*1*_ *+ β*_*3*_) = 0.996 *P*-value = 0.012).

**Table 2:** Parameter estimates, standard errors and P-values from the segmented regression models predicting the outcomes

	Exp(β)	Exp(SE(β))	95% Confidence interval	*P*-value
**A. Marriage rates**				
Intercept *β*_*0*_	0.000	1.057	(0.000–0.000)	< 0.0001
Baseline outcome trend *β*_*1*_	0.997	1.004	(0.990–1.003)	0.314
Level change after intervention *β*_*2*_	1.046	1.082	(0.896–1.222)	0.566
Trend change after intervention *β*_*3*_	1.002	1.005	(0.992–1.012)	0.740
Follow-up outcome trend *β*_*1*_ *+ β*_*3*_	0.998	1.004	(0.991–1.005)	0.606
**B. Pregnancy rates**				
Intercept *β*_*0*_	0.000	1.024	(0.000–0.000)	< 0.0001
Baseline outcome trend *β*_*1*_	0.993	1.002	(0.990–0.996)	< 0.0001
Level change after intervention *β*_*2*_	0.987	1.035	(0.921–1.057)	0.709
Trend change after intervention *β*_*3*_	1.002	1.002	(0.998–1.007)	0.279
Follow-up outcome trend *β*_*1*_ *+ β*_*3*_	0.996	1.002	(0.992–0.999)	0.008
** C. Multiple pregnancy rates**				
Intercept *β*_*0*_	0.000	0.033	(0.000–0.000)	< 0.0001
Baseline outcome trend *β*_*1*_	0.994	0.002	(0.990–0.999)	0.007
Level change after intervention *β*_*2*_	1.025	0.047	(0.935–1.123)	0.603
Trend change after intervention *β*_*3*_	1.010	0.003	(1.005–1.016)	0.001
Follow-up outcome trend *β*_*1*_ *+ β*_*3*_	1.005	0.002	(1.001–1.009)	0.025
**D. Multiple birth rates**				
Intercept *β*_*0*_	0.000	0.039	(0.000–0.000)	< 0.0001
Baseline outcome trend *β*_*1*_	0.991	0.003	(0.986–0.996)	0.0003
Level change after intervention *β*_*2*_	0.978	0.059	(0.872–1.097)	0.706
Trend change after intervention *β*_*3*_	1.014	0.004	(1.007–1.022)	< 0.0001
Follow-up outcome trend *β*_*1*_ *+ β*_*3*_	1.005	0.003	(1.000–1.010)	0.054
**E. Total birth rates**				
Intercept *β*_*0*_	0.000	1.024	(0.000–0.000)	< 0.0001
Baseline outcome trend *β*_*1*_	0.993	1.002	(0.990–0.996)	< 0.0001
Level change after intervention *β*_*2*_	0.986	1.036	(0.921–1.057)	0.699
Trend change after intervention *β*_*3*_	1.003	1.002	(0.998–1.007)	0.237
Follow-up outcome trend *β*_*1*_ *+ β*_*3*_	0.996	1.002	(1.000–1.000)	0.012

**Fig. 1:**
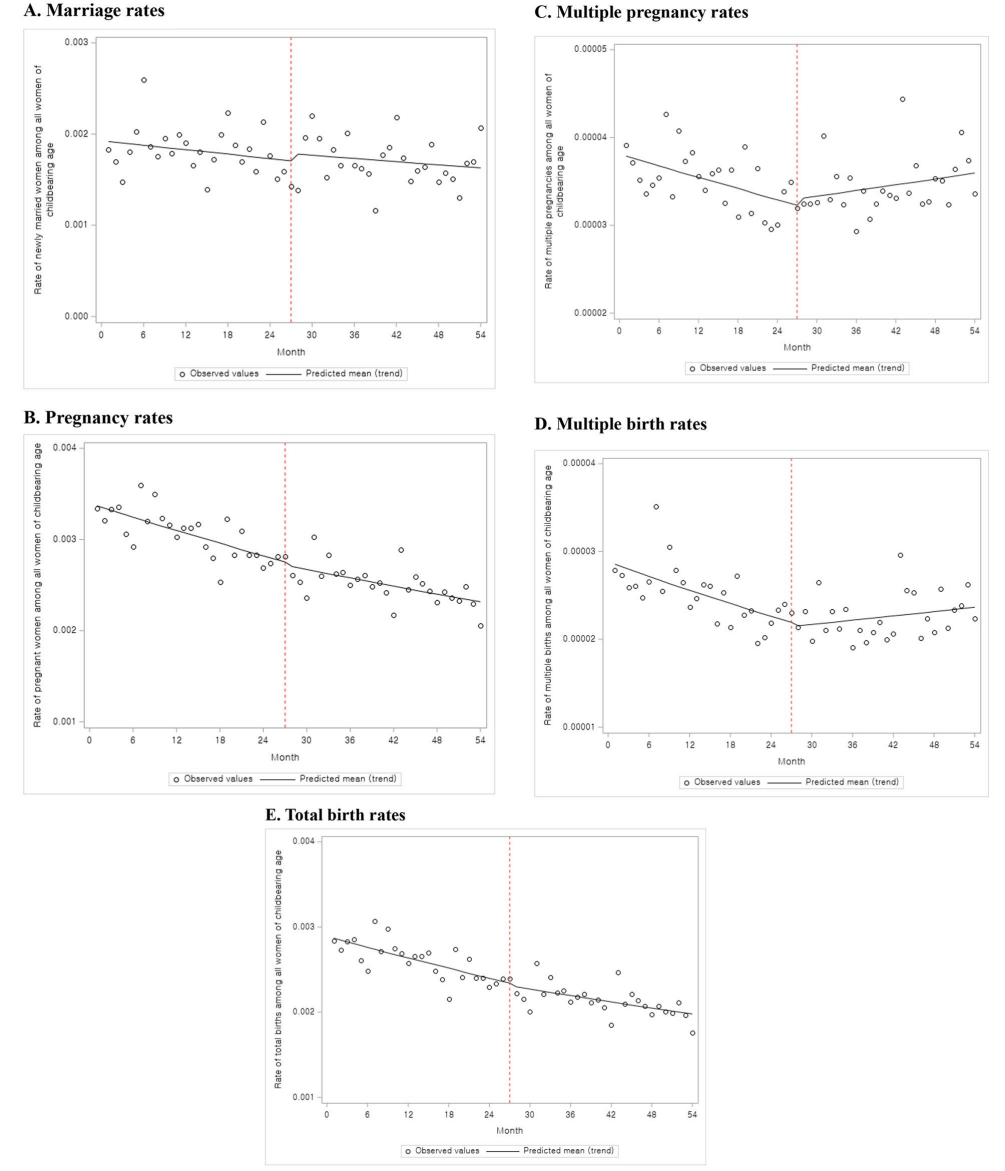
ITS results for the rates of marriage, pregnancy, multiple pregnancy, multiple births, and total birth

Table 2 provides quantitative confirmation of the size of the level and trend changes through parameter estimates, whereas Fig. 1 provides an intuitive confirmation of the outcome trend after the intervention. The ITS analysis revealed an increase in multiple pregnancy and birth rates. Additionally, although the pregnancy rates and total birth rates continue to decrease, it can be intuitively confirmed the slope has become slightly gentler after the intervention.

## Discussion

This study aims to assess the impact of introducing ART health insurance as a remedy for the low fertility rate in Korea, which is currently the lowest in the world. The primary results of this study are as follows. ART had no noticeable effect on marriage or pregnancy rates. However, following the implementation of the policy, there was a marked increase in both multiple pregnancy and birth rates, with the trend shifting from declining to increasing. Additionally, no effect of ART coverage on the national birth rate was observed; however, the decline significantly flattened after the intervention. To our knowledge, health insurance coverage for ART positively contributes to the rates of multiple pregnancies and births. While it was not completely covered, and patients were required to pay 30% of the total treatment cost, this intervention had a significant effect. As the decline in the total birth rate slows, it is expected that additional interventions will contribute to increasing the total birth rate in the long term.

Most previous studies on the impact of ART have primarily focused on evaluating the economic burden [16, 17] and utilization of ART treatment [18] from the patient’s perspective. These studies show that implementing ART coverage policies can reduce patient costs and increase ART utilization. However, few studies have examined the effects of ART coverage policies on fertility improvement from a social perspective and only a few have examined the effects of ART on maternal health outcomes. They reported that mothers who underwent ART had a higher risk of severe maternal morbidities [19], and children born with ART may have poorer health outcomes and a higher risk of developing certain diseases [20, 21]. Despite these findings, other studies suggest that ART contributes to improved fertility rates and demographic changes [22]. Their results aligned with the findings of our study; it is essential to promote ART as a nationwide approach to address the issue of declining fertility rates.

This study evaluated the nationwide impact of the ART treatment coverage policy, which is important because the high cost of ART treatment places a significant financial burden on infertile couples [17]. Korea’s NHIS has been providing 70% coverage of ART treatment costs since October 2017, which is the first time such coverage has become available for those with above-median incomes. This policy has led to an increase in multiple pregnancies and births and may slow the decline in Korea’s total birth rate. The introduction of additional interventions, such as ART, to support infertile couples is expected to solve the problems of low birth rates and population decline.

Nevertheless, Korea’s policy, which allows for an unlimited number of embryos, could be enhanced when compared to numerous other developed countries that adopt a single embryo transfer policy [23–25]. This is because these countries, even if they provide public funding for ART treatment, do not aim to increase the number of births. Not placing restrictions on the number of embryos during ART procedures, as seen in Korea, can result in the occurrence of multiple births and a rise in live births. However, this approach can adversely affect maternal and fetal health outcomes. Therefore, when it comes to publicly funded ART, Korea also needs to consider a policy of limiting the number of transplanted embryos in order to protect maternal and child health.

This study has certain limitations. First, as we analyzed aggregated data from Statistics Korea, it was not feasible to assess the policy’s impact on individual patients. Second, one drawback of using administrative claims data is that the disease codes recorded in the NHIS cohort dataset might not accurately represent the participants’ actual health conditions because these codes were generated for the billing health insurance of the participants. Third, we had no control over all other informal benefits and interventions available to infertile couples at the time ART insurance coverage was introduced. While autocorrelation due to external policy effects may be concern, the insurance mandates for ART treatment was certainly the most comprehensive and formal intervention promoted at the time. Nevertheless, this study has multiple strengths. We used representative national statistical data, including the entire population, for the calculation of the outcome variables; therefore, our results can serve as an important basis for evaluating the effects of policies at the national level. Additionally, we used an ITS design, which is a strong quasi-experimental approach for evaluating the longitudinal effects of interventions [26]. The advantage of this design lies in its ability to fully use the longitudinal nature of the data and consider pre-intervention trends [14]. Some prior studies have employed a difference-in-differences design that only compares two time points to examine the net policy impact on the outcomes [27], or segmented regression with fewer than 10 time points [28]. As there were insufficient time points to capture baseline trends and outcome changes, this study used 54 time points (27 time points each before and after the intervention) to capture changes in trends more robustly.

## Conclusions

Our findings showed that the ART health insurance coverage policy led to higher rates of multiple pregnancies and births. Additionally, although the total birth rate has been continuously decreasing over time, the decline may have been slowed down slightly by this policy. To overcome the serious problems of low birth rates and population decline, policies supporting infertile couples should be developed and implemented.

## Electronic supplementary material

Below is the link to the electronic supplementary material.

Supplementary Material 1

## Data Availability

The data is publicly accessible on the website of MicroData Integrated Service (MDIS) operated by Statistics Korea (https://mdis.kostat.go.kr/), and the website of Korean National Health Insurance Sharing Service (NHISS) operated by Korean NHIS (https://nhiss.nhis.or.kr/).

## Declarations

the study protocol was reviewed and approved by the Institutional Review Board of Eulji University (IRB Number: EU22-27).

## List of abbreviations

ITS: Interrupted time series
NHIS: National Health Insurance Service
OECD: Organization for Economic Cooperation and Development
TFR: Total fertility rate
UN: United Nations
