# Supplementary material for: The impact of assisted reproductive technology treatment coverage on marriage, pregnancy, and childbirth in women of childbearing age: an interrupted time-series analysis
Source: BMC Public Health. 2023 Jul 18;23:1379. doi: 10.1186/s12889-023-16286-3 (PMC10353198; doi:10.1186/s12889-023-16286-3)
Supplement: Supplementary file 1 — Supplementary Material 1 [file 12889_2023_16286_MOESM1_ESM.docx]

| **Supplementary Table 1. Structure of aggregated data for analysis of the impact of intervention on marriage rates, pregnancy rates, multiple pregnancy rates, multiple birth rates, and total birth rates** | | | | | | | | | |
| --- | --- | --- | --- | --- | --- | --- | --- | --- | --- |
| **Year_date** | **Number of women of childbearing age** | **TIME (month)** | **Intervention (ART health coverage)** | **Time after intervention** | **Number of newly married women** | **Number of pregnant women** | **Number of mothers with multiple pregnancy** | **Number of mothers with multiple birth** | **Total number of births** |
| 2015.07 | 12,882,830 | 1 | 0 | 0 | 23,571 | 43,033 | 504 | 359 | 36,612 |
| 2015.08 | 12,873,152 | 2 | 0 | 0 | 21,821 | 41,364 | 478 | 352 | 35,207 |
| 2015.09 | 12,865,036 | 3 | 0 | 0 | 19,001 | 42,894 | 452 | 333 | 36,444 |
| 2015.10 | 12,855,867 | 4 | 0 | 0 | 23,237 | 43,198 | 432 | 335 | 36,702 |
| 2015.11 | 12,847,833 | 5 | 0 | 0 | 26,047 | 39,357 | 444 | 318 | 33,467 |
| 2015.12 | 12,831,407 | 6 | 0 | 0 | 33,294 | 37,434 | 454 | 341 | 31,910 |
| 2016.01 | 12,824,873 | 7 | 0 | 0 | 23,869 | 46,164 | 547 | 451 | 39,405 |
| 2016.02 | 12,810,376 | 8 | 0 | 0 | 22,474 | 40,971 | 426 | 327 | 34,830 |
| 2016.03 | 12,789,222 | 9 | 0 | 0 | 24,991 | 44,781 | 521 | 390 | 38,131 |
| 2016.04 | 12,780,991 | 10 | 0 | 0 | 22,844 | 41,282 | 477 | 356 | 35,147 |
| 2016.05 | 12,773,701 | 11 | 0 | 0 | 25,489 | 40,358 | 489 | 338 | 34,341 |
| 2016.06 | 12,770,265 | 12 | 0 | 0 | 24,285 | 38,652 | 454 | 303 | 32,849 |
| 2016.07 | 12,763,899 | 13 | 0 | 0 | 21,154 | 39,908 | 434 | 315 | 33,920 |
| 2016.08 | 12,755,163 | 14 | 0 | 0 | 23,017 | 39,832 | 458 | 335 | 33,897 |
| 2016.09 | 12,746,767 | 15 | 0 | 0 | 17,763 | 40,413 | 463 | 332 | 34,375 |
| 2016.10 | 12,736,136 | 16 | 0 | 0 | 21,951 | 37,203 | 414 | 278 | 31,592 |
| 2016.11 | 12,728,724 | 17 | 0 | 0 | 25,385 | 35,624 | 462 | 323 | 30,366 |
| 2016.12 | 12,715,603 | 18 | 0 | 0 | 28,413 | 32,175 | 394 | 272 | 27,390 |
| 2017.01 | 12,697,636 | 19 | 0 | 0 | 23,845 | 40,930 | 495 | 346 | 34,834 |
| 2017.02 | 12,669,289 | 20 | 0 | 0 | 21,501 | 35,865 | 398 | 289 | 30,499 |
| 2017.03 | 12,644,701 | 21 | 0 | 0 | 23,290 | 39,090 | 461 | 294 | 33,196 |
| 2017.04 | 12,631,387 | 22 | 0 | 0 | 20,076 | 35,772 | 383 | 247 | 30,337 |
| 2017.05 | 12,618,845 | 23 | 0 | 0 | 26,924 | 35,712 | 373 | 255 | 30,303 |
| 2017.06 | 12,609,921 | 24 | 0 | 0 | 22,292 | 33,968 | 379 | 276 | 28,892 |
| 2017.07 | 12,598,755 | 25 | 0 | 0 | 18,964 | 34,556 | 427 | 294 | 29,418 |
| 2017.08 | 12,584,383 | 26 | 0 | 0 | 20,068 | 35,397 | 439 | 302 | 30,135 |
| 2017.09 | 12,570,884 | 27 | 0 | 0 | 17,933 | 35,366 | 402 | 290 | 30,085 |
| 2017.10 | 12,554,539 | 28 | 1 | 1 | 17,348 | 32,760 | 407 | 268 | 27,857 |
| 2017.11 | 12,538,776 | 29 | 1 | 2 | 24,629 | 31,758 | 407 | 291 | 27,068 |
| 2017.12 | 12,516,864 | 30 | 1 | 3 | 27,585 | 29,556 | 408 | 248 | 25,147 |
| 2018.01 | 12,497,178 | 31 | 1 | 4 | 24,370 | 37,818 | 502 | 331 | 32,198 |
| 2018.02 | 12,474,297 | 32 | 1 | 5 | 19,008 | 32,434 | 411 | 263 | 27,575 |
| 2018.03 | 12,450,673 | 33 | 1 | 6 | 22,773 | 35,266 | 443 | 289 | 29,987 |
| 2018.04 | 12,438,287 | 34 | 1 | 7 | 20,610 | 32,622 | 403 | 264 | 27,734 |
| 2018.05 | 12,424,580 | 35 | 1 | 8 | 24,996 | 32,815 | 440 | 291 | 27,949 |
| 2018.06 | 12,413,824 | 36 | 1 | 9 | 20,610 | 31,035 | 364 | 237 | 26,357 |
| 2018.07 | 12,390,783 | 37 | 1 | 10 | 20,091 | 31,788 | 420 | 261 | 27,033 |
| 2018.08 | 12,371,215 | 38 | 1 | 11 | 19,345 | 32,249 | 380 | 243 | 27,381 |
| 2018.09 | 12,351,096 | 39 | 1 | 12 | 14,344 | 30,632 | 401 | 257 | 26,066 |
| 2018.10 | 12,328,913 | 40 | 1 | 13 | 21,856 | 31,088 | 418 | 271 | 26,474 |
| 2018.11 | 12,314,765 | 41 | 1 | 14 | 22,801 | 29,741 | 412 | 246 | 25,301 |
| 2018.12 | 12,294,729 | 42 | 1 | 15 | 26,818 | 26,681 | 407 | 254 | 22,767 |
| 2019.01 | 12,272,101 | 43 | 1 | 16 | 21,326 | 35,424 | 545 | 363 | 30,271 |
| 2019.02 | 12,247,619 | 44 | 1 | 17 | 18,199 | 30,071 | 413 | 313 | 25,710 |
| 2019.03 | 12,224,263 | 45 | 1 | 18 | 19,549 | 31,685 | 450 | 310 | 27,049 |
| 2019.04 | 12,205,699 | 46 | 1 | 19 | 20,026 | 30,714 | 396 | 246 | 26,104 |
| 2019.05 | 12,186,232 | 47 | 1 | 20 | 23,045 | 29,684 | 398 | 273 | 25,299 |
| 2019.06 | 12,173,264 | 48 | 1 | 21 | 17,942 | 28,168 | 430 | 253 | 23,992 |
| 2019.07 | 12,155,429 | 49 | 1 | 22 | 19,178 | 29,500 | 427 | 313 | 25,222 |
| 2019.08 | 12,136,190 | 50 | 1 | 23 | 18,336 | 28,609 | 393 | 259 | 24,371 |
| 2019.09 | 12,118,848 | 51 | 1 | 24 | 15,798 | 28,214 | 441 | 283 | 24,090 |
| 2019.10 | 12,100,735 | 52 | 1 | 25 | 20,327 | 30,027 | 491 | 289 | 25,613 |
| 2019.11 | 12,083,809 | 53 | 1 | 26 | 20,488 | 27,697 | 452 | 317 | 23,727 |
| 2019.12 | 12,065,205 | 54 | 1 | 27 | 24,945 | 24,821 | 405 | 270 | 21,228 |
